# Effects of emotional regulation and impulsivity on sports performance: the mediating role of gender and competition level

**DOI:** 10.3389/fpsyg.2023.1164956

**Published:** 2023-07-03

**Authors:** Antonio Millán-Sánchez, Iker Madinabeitia, Ricardo de la Vega, David Cárdenas, Aurelio Ureña

**Affiliations:** ^1^Department of Physical Education and Sport, University of Granada, Granada, Spain; ^2^Department of Physical Education, Sport and Human Movement, Autonomous University of Madrid, Madrid, Spain

**Keywords:** emotional regulation, impulsivity, sport performance, gender, competition level, volleyball, playing position

## Abstract

**Introduction:**

This study aimed to study the relationships between emotional regulation and impulsivity on sports performance, according to the gender and competition level in national-level volleyball players.

**Methods:**

In total, 77 players from the 2018–2019 top two Spanish divisions completed the ERQ (emotion regulation) and the UPPS-P (impulsivity). Statistics (serve, reception, and attack) were retrieved from the Spanish Volleyball Federation. A Mann–Whitney test was conducted to determine differences between gender and competition level in impulsivity and emotion regulation. A Kruskal–Wallis test (Mann–Whitney *post-hoc*) was carried out for playing positions analysis. Spearman's correlation was performed between the performance and the variables of impulsivity and emotional regulation.

**Results:**

The results obtained, with differences according to playing position, gender, and competition level, show that players differ in certain psychological aspects that might influence how they approach their game.

**Discussion:**

The results point in a promising direction: the sports practice analyzed can serve as a regulation context, for both the emotional response and the level of impulsivity of the players. The importance of these results for future research on this topic is discussed.

## Introduction

To achieve successful results in sports, coaches commonly evaluate technical, tactical, or physical aspects. Playing volleyball entails constant short-term decision-making, by which, depending on the time available, athletes process the environmental information consciously or unconsciously (Acker, 2008). Thus, it is also advisable to consider emotional aspects in this analysis, such as the strategies they follow to regulate their emotions and impulsivity (Lage et al., 2011; Castillo-Rodríguez et al., 2018; Krenn et al., 2018).

In sports, decisions are frequently unconscious and influenced by essential or incidental emotions (Qiu, 2015). Essential emotions derive from the direct practice in the task (i.e., a mistake during the game might cause a decrease in the confidence of a player), whereas incidental emotions are generated by factors outside the task but interact with essential ones during the decision-making (i.e., a sentimental breakup might lead to poor performance). Therefore, emotion regulation (ER) is a relevant field of study in sports. ER can be defined as the mechanism through which individuals modify (either intentionally or unintentionally) their emotions to achieve the desired outcome (Aldao et al., 2010). According to the model

by Gross and John (2003), suppression is one of the possible ER strategies and refers to how people inhibit their expressive answers. The other ER strategy is cognitive reappraisal, which refers to how people try to alter the emotional impact caused by the situation or context. Given the stressful situations that can arise during a volleyball match (i.e., technical or tactical mistakes that mean a loss of points for the whole team), how players cope with anxiety and manage their ER strategies represent a relevant avenue of research. To the best of our knowledge, no studies concerning ER have been conducted on volleyball players, although its possible relationship with sports has been observed in other studies (Cárdenas et al., 2018).

Recent studies have found a correlation between some variables of the ER (i.e., suppression) and some dimensions of impulsivity, precisely negative urgency, and lack of premeditation (Wypych et al., 2018; Jara-Rizzo et al., 2019). Moeller et al. (2001) defined *impulsivity* as “a predisposition toward rapid, unplanned reactions to internal or external stimuli without regard to the negative consequences of these reactions to the impulsive individual or others” (p. 1784). Impulsivity affects performance in tasks when control is needed, and this interference can be increased in situations of decision-making (Stanford et al., 2009). Precisely by the characteristics impulsivity as a personality trait, high-level volleyball has been selected, where rallies last 5 s on average (Sánchez-Moreno et al., 2015). Therefore, points are won or lost based on quick decisions, most unconscious, generating multiple emotional responses that involve control and regulation (Acker, 2008). The impulsivity might interfere somehow with the result of those decisions. Some studies have dealt with the relationship between playing positions, impulsivity, and performance in sports, namely futsal (Castillo-Rodríguez et al., 2018) or handball (Lage et al., 2011). However, we have not found studies performed on a volleyball sample.

Due to the possible influence of game roles within the emotional experience of athletes, it has been decided to also study the possible differential effect of this variable on ER and impulsivity. It has been widely demonstrated that the playing position is a relevant factor to take into account when studying tactical, technical, or physical differences in volleyball (Sheppard et al., 2009; Lobietti et al., 2010; Millán-Sánchez et al., 2015). Thus, it is plausible to consider that differences may also appear in emotional variables, making their mental approach different too (do Nascimento et al., 2016). In other team sports, it has previously been demonstrated that there is a correlation between some dimensions of impulsivity (i.e., sensation seeking) and the player's performance during the game (Castillo-Rodríguez et al., 2018). This type of results leads us to consider that these relationships might also be found in volleyball, providing valuable information that coaches could use to achieve higher performance for their players.

Regarding gender, the differences in impulsivity are hard to explain. As Cross et al. (2011) reflect, higher impulsivity in male individuals might have an evolutionary reason, but they do not present a comparison with female individuals. Research on the general population has shown contradictory results (Marazziti et al., 2010; Lage et al., 2013). It might be interesting to check whether any differences appear in volleyball players, given that studies on athlete samples have shown no differences so far (Kotnik et al., 2012; Ferreira et al., 2017). Concerning the competition level, not much research has been conducted. Studies have usually focused on risk athletes (i.e., rock climbing, free diving, or snowboarding), comparing between experts and beginners, or even between athletes and non-athletes. As with gender, different conclusions have been reported (Thomson and Carlson, 2014; Dudek et al., 2016).

Therefore, considering the current lack of research on this topic, this study aimed to study the relationships between ER, impulsivity, and individual performance, according to the playing position, gender, and competition level in high-level volleyball during the 2018–2019 season in professional players from the top Spanish volleyball leagues (first division and second division). How do these variables relate to the players' performance, playing position, gender, and level of competition? The authors hypothesized that they could be related to individual performance depending on the playing position, gender, and level of competition. In addition, it is suggested that players with a higher competitive level regulate their emotions better and have lower levels of impulsivity.

## Materials and methods

### Participants and ethical approval

In total, 77 volleyball players [mean ± SD: 24.4 ± 4.3 years old; 31 male players, 46 female players; 28 first division players (13 male players, 15 female players; 26.3 ± 4.4 years old), 49 second division players (18 male players, 31 female players; 23.3 ± 3.9 years old)] from the top two Spanish divisions took part in the study. All of them were fluent in Spanish (although some of them were from different countries). The data were collected confidentially after the competitive season's ending under the Institutional Review Board guidelines of the University of Granada (IRB Approval No. 731/CEIH/2018) and following the Declaration of Helsinki (2013). Informed consent was obtained from all participants.

### Instruments and materials

#### Emotional regulation

In order to assess both ER strategies, Gross and John (2003) developed the emotion regulation questionnaire (ERQ). Uphill et al. (2012) checked the validity of this instrument within a sports sample. The Spanish version of the ERQ was used (Cabello et al., 2013). It consists of 10 items that assess cognitive reappraisal (six items; e.g., “When I'm faced with a stressful situation, I make myself think about it in a way that helps me stay calm”; α = 0.82) and suppression (four items; e.g., “I keep my emotions to myself”; α = 0.74). The scale is a 7-point Likert score from 1 (strongly disagree) to 7 (strongly agree). The sum of each strategy was calculated.

#### Impulsivity

A standard tool to measure impulsivity is the UPPS-P (Lynam et al., 2006). It assesses five dimensions: negative urgency (NU, an act of rash under negative emotions; e.g., “When I am upset, I often act without thinking”; α = 0.79), positive urgency (PU, an act of rash under positive emotions; e.g., “I tend to lose control when I am in a great mood”; α = 0.69), lack of premeditation (LPr, the tendency to disregard the consequences of actions; e.g., “My thinking is usually careful and purposeful”; α = 0.71), lack of perseverance (LPe, the tendency to leave tasks unfinished; e.g., “I finish what I start”; α = 0.69), and sensation seeking (SS, the predisposition to new experiences; e.g., “I quite enjoy taking risks”; α = 0.74 (Whiteside and Lynam, 2001). The Spanish short version of the UPPS-P was used (Cándido et al., 2012). It comprises 20 items scored on a 4-point Likert scale, from 1 (strongly agree) to 4 (strongly disagree). There are 4 items for each dimension. The scores NU, PU, and SS were reversed. The sum of each dimension was calculated. A higher sum represents greater impulsivity.

#### Performance indicators

The player statistics (data regarding serve, reception, and attack) during the 2018/2019 season were collected from the Spanish Volleyball Federation (www.rfevb.com). Certified operators register the statistics by the Spanish Volleyball Federation, who are instructed to follow the same criteria to obtain reliable information, so it is assumable that the collected data fulfill the required reliability. Based on the performance, liberos and middle blockers cannot be compared, whereas setters and wing spikers share one common action (serve). With this in mind, only the differences between wing spikers and middle blockers can be explained by comparing their performance in two actions. Therefore, three performance variables for serve and attack combined were calculated: error ratio (Equation 1), efficacy (Equation 2), and efficiency (Equation 3).


$$\begin{array}{l} Error\;ratio\;=\;\frac{serve\;errors\;+\;attack\;errors}{total\;serves\;+\;total\;attacks} \end{array}$$

Equation 1. Formula to calculate the error ratio.


$$\begin{array}{l} Efficacy\;=\;\frac{serve\;points\;+\;attack\;points}{total\;serves\;+\;total\;attacks} \end{array}$$

Equation 2. Formula to calculate the efficacy.


$$\begin{array}{l} \begin{array}{l} Efficiency\;\\ \;=\;\frac{\left(serve\;points\;+\;attack\;points)\;-\;(serve\;errors\;+\;attack\;errors\right)}{total\;serves\;+\;total\;attacks} \end{array} \end{array}$$

Equation 3. Formula to calculate the efficiency.

#### Playing position

The players in the questionnaire stated their playing positions, and it was divided into 4: setters (15), wing spikers (39), middle blockers (19), and liberos (14). This classification is an adaptation from the one used by Millán-Sánchez et al. (2015).

### Procedure

The staff of 16 volleyball teams of the top two Spanish divisions were contacted to participate in this study. In total, 10 of those teams responded positively and were visited on their usual training session schedule, after the competitive season, in an interval of 4 weeks. The players were informed about the study and instructed on how to complete the ERQ and the UPPS-P questionnaires, both in Spanish. The participants signed the informed consent forms and completed the questionnaires before their training session. The playing positions (setter, wing spiker, middle blocker, or libero), gender (male or female), and category (first or second division) of the players were stated in the questionnaires.

### Statistical analysis

Data summaries (mean and standard deviation) were computed for the whole sample. There were no missing values. Outliers (between 0 and 3.9%, depending on the variable) were not removed in order not to decrease the sample size. Shapiro–Wilk normality tests showed that the ER and impulsivity variables did not present a normal distribution. Therefore, non-parametric analyses were conducted. A Mann–Whitney test was conducted to determine differences between gender and competition level in ER and impulsivity. The effect size was calculated with *r*, whose formula is z/√(n), where *z* is the z-statistic and *n* is the number of observations. Its interpretation follows the Cohen guidelines (Cohen, 1988): 0.10 represents a small association, 0.30 moderate association, and 0.50 large association. For playing positions analysis, a Kruskal–Wallis test was carried out. Effect sizes were calculated with the Epsilon-squared coefficient (*E_R_^2^*; Kelley, 1935), following the interpretations mentioned above.

Moreover, when significant results were observed, a U of Mann–Whitney *post-hoc* was conducted to determine the groups significantly different, with their respective effect size (*r*). Finally, Spearman's correlation (ρ) was performed between the variables of performance and the variables of ER and impulsivity in order to check their relationship. The level of significance was established at 0.05 for all the analyses. The statistical instrument used was SPSS for Windows, version 22.0 (IBM Corp., Armonk, NY) and JASP version 0.10 (JASP team, Amsterdam).

## Results

### Descriptive results

#### ER and impulsivity according to the playing position

The data concerning the playing position are presented in Table 1. Regarding ER, players tended to use a strategy based on reappraisal more than suppression. Importantly, liberos showed the lowest values in both ER strategies, whereas middle blockers presented the highest scores in reappraisal and wing spikers in suppression. Concerning impulsivity, the lowest scores were found in the dimension of LPe and the highest in SS. Liberos presented the highest values in NU, LPr, and LPe, whereas wing spikers were in SS and PU. Setters presented the lowest values in LPr, LPe, and PU, whereas middle blockers were in NU and SS.

**Table 1:** Descriptive statistics (mean ± SD) of the variables of the ERQ and UPPS-P, according to the playing position.

	**Playing position**			
	**Setter**	**Wing spiker**	**Middle blocker**	**Libero**
Reappraisal	29.62 ± 7.89	27.44 ± 7.17	30.59 ± 4.66	28.09 ± 6.98
Suppression	14.69 ± 5.36	15.11 ± 5.81	14.29 ± 5.42	14.18 ± 5.47
NU	7.62 ± 2.87	9.81 ± 2.64	7.12 ± 2.12	10.20 ± 3.33
LPr	6.85 ± 2.12	7.33 ± 2.07	7.47 ± 1.77	7.50 ± 1.90
LPe	6.23 ± 1.74	6.44 ± 1.92	6.41 ± 1.97	6.50 ± 1.18
SS	11.23 ± 2.62	11.83 ± 2.35	10.23 ± 2.59	11.3 ± 2.11
PU	9.08 ± 3.01	10.25 ± 1.92	9.53 ± 2.21	10.20 ± 2.94

NU, Negative urgency; LPe, lack of perseverance; LPr, lack of premeditation; SS, sensation seeking; PU, positive urgency.

#### ER and impulsivity according to the gender and competition level

The descriptive data regarding gender and competition level are presented in Table 2. Male players presented a higher reappraisal strategy than female players, whereas the latter showed higher suppression than the former. Similarly, 1st division players seemed to use an ER strategy based on suppression more than second division players, whereas the latter showed a tendency toward reappraisal higher than the former. Concerning impulsivity, men presented higher sums in LPr and LPe, and women in NU, SS, and PU. First division players presented higher values in NU and second division players in all the other dimensions.

**Table 2:** Descriptive statistics (mean ± SD) of the variables of the variables of the ERQ and UPPS-P, according to the gender and the competition level.

	**Gender**		**Competition level**	
	**Male**	**Female**	**First division**	**Second division**
Reappraisal	4.99 ± 0.98	4.61 ± 1.21	4.73 ± 1.14	4.79 ± 1.14
Suppression	3.60 ± 1.48	3.74 ± 1.32	3.80 ± 1.40	3.61 ± 1.38
NU	7.87 ± 2.64	9.54 ± 2.92	9.15 ± 3.12	8.73 ± 2.81
LPr	7.50 ± 1.82	7.17 ± 2.07	7.07 ± 1.82	7.43 ± 2.05
LPe	6.80 ± 1.67	6.15 ± 1.84	6.15 ± 2.05	6.55 ± 1.63
SS	10.97 ± 2.16	11.52 ± 2.63	10.44 ± 2.26	11.78 ± 2.45
PU	9.67 ± 2.55	10.02 ± 2.21	9.63 ± 2.45	10.02 ± 2.29

NU, Negative urgency; LPe, lack of perseverance; LPr, lack of premeditation; SS, sensation seeking; PU, positive urgency.

### Performance associations

The collected statistics provide information about the serve, reception, and attack. Therefore, liberos and middle blockers cannot be compared based on performance, whereas setters and wing spikers share one common action (serve). Given these results, only the differences between wing spikers and middle blockers can be explained by comparing their performance in two actions. Thus, we calculated the error ratio, efficacy, and efficiency for serve and attack altogether and correlated it to the different dimensions of impulsivity (Table 3). A correlation was performed between ER, impulsivity, and the performance indicators. The error ratio correlated positively with the NU (ρ = 0.276, *p* < 0.05) and with the SS (ρ = 0.302, *p* < 0.05). No significant correlations were found with the remaining dimensions of impulsivity (LPr, LPe, and PU) and the variables of ER (reappraisal and suppression). The correlation with the NU was small and with the SS was medium, according to Cohen (1988). The efficacy and efficiency did not correlate with any dimension of impulsivity.

**Table 3:** Spearman's correlation values of the error ratio, the efficacy, and the efficiency with the ER strategies (reappraisal and suppression) and the dimensions of the impulsivity (negative urgency, lack of premeditation, lack of perseverance, sensation seeking, and positive urgency).

	**Error ratio**		**Efficacy**		**Efficiency**	
	**Spearman's rho**	***P***	**Spearman's rho**	***P***	**Spearman's rho**	***P***
Reappraisal	−0.197	0.158	−0.084	0.549	−0.069	0.623
Suppression	0.057	0.686	−0.015	0.915	−0.053	0.706
NU	0.276^*^	0.046	−0.083	0.554	−0.183	0.191
LPr	0.137	0.329	−0.076	0.591	−0.117	0.404
LPe	0.173	0.217	−0.113	0.421	−0.207	0.137
SS	0.302^*^	0.028	0.066	0.639	−0.090	0.524
PU	0.047	0.736	−0.075	0.592	−0.110	0.433

^*^p < 0.05.

NU, Negative urgency; LPe, lack of perseverance; LPr, lack of premeditation; SS, Sensation seeking; PU, Positive urgency.

### Differences between playing positions

A Kruskal–Wallis test was conducted for all the dependent variables (Table 4). Results showed no statistical differences for the ER strategies (reappraisal and suppression). Only one dimension of impulsivity (NU) showed significance (χ^2^ = 14.826; *p* = 0.002; *E_R_^2^* = 0.20) according to the playing position.

**Table 4:** Kruskal–Wallis test for the variables of the ERQ (reappraisal and suppression) and the UPPS-P (NU, LPr, LPe, SS, and PU), according to the playing position.

	**χ^2^**	***p***	***E_R_^2^***
Reappraisal	3.370	0.338	0.04
Suppression	0.427	0.935	0.01
NU	14.826	0.002	0.20
LPr	0.798	0.850	0.01
LPe	0.124	0.989	0.00
SS	4.786	0.188	0.06
PU	3.882	0.274	0.05

χ^2^, Chi-squared; E${}_{\text{R}}^{2}$, epsilon-squared.

**Figure 1:**
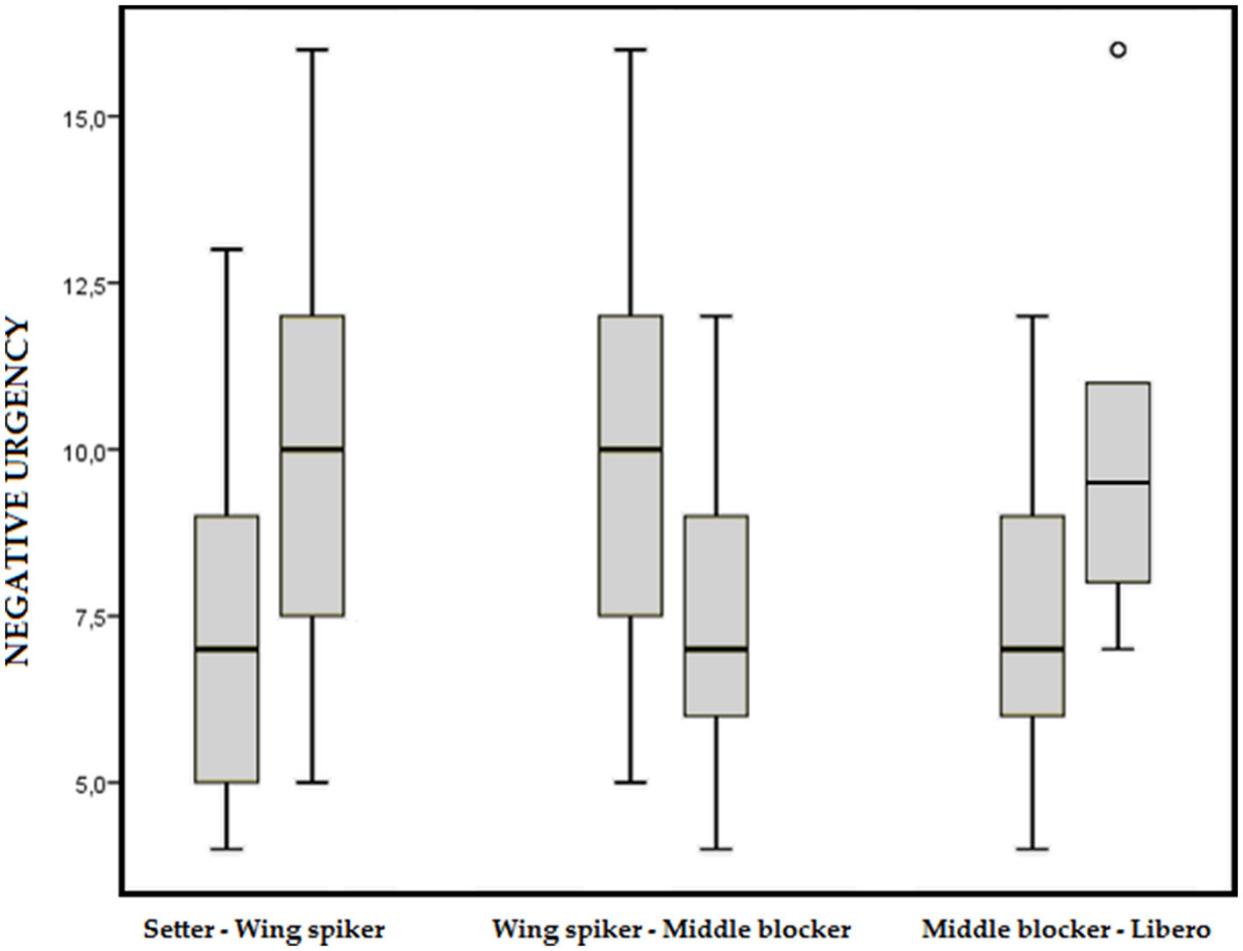
Comparison of the significant pairwise differences for NU according to the playing position.

A *post-hoc U* of Mann–Whitney was conducted to assess pairwise differences for NU (Figure 1). Significant differences were found between setters and wing spikers (*Z* = 2.324; *p* = 0.02; *r* = 0.33), wing spikers and middle blockers (*Z* = 3.348; *p* = 0.001; *r* = 0.46), and middle blockers and liberos (*Z* = 2.607; *p* = 0.009; *r* = 0.50).

### Differences between gender and competition level

**Figure 2:**
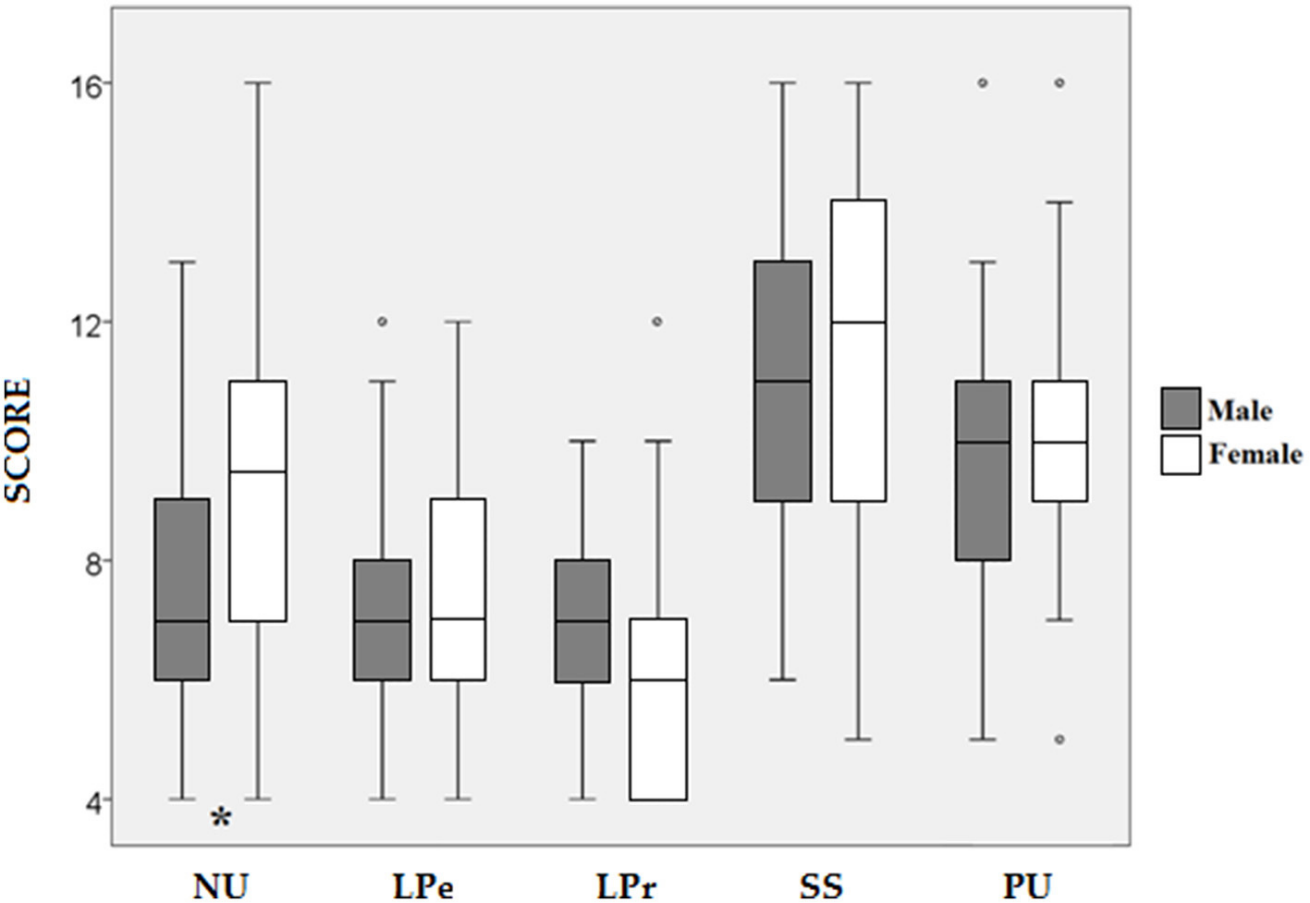
Comparison of the scores obtained in the dimensions of impulsivity according to gender. NU, Negative urgency; LPe, lack of perseverance; LPr, lack of premeditation; SS, sensation seeking; PU, positive urgency. *p < 0.05.

**Figure 3:**
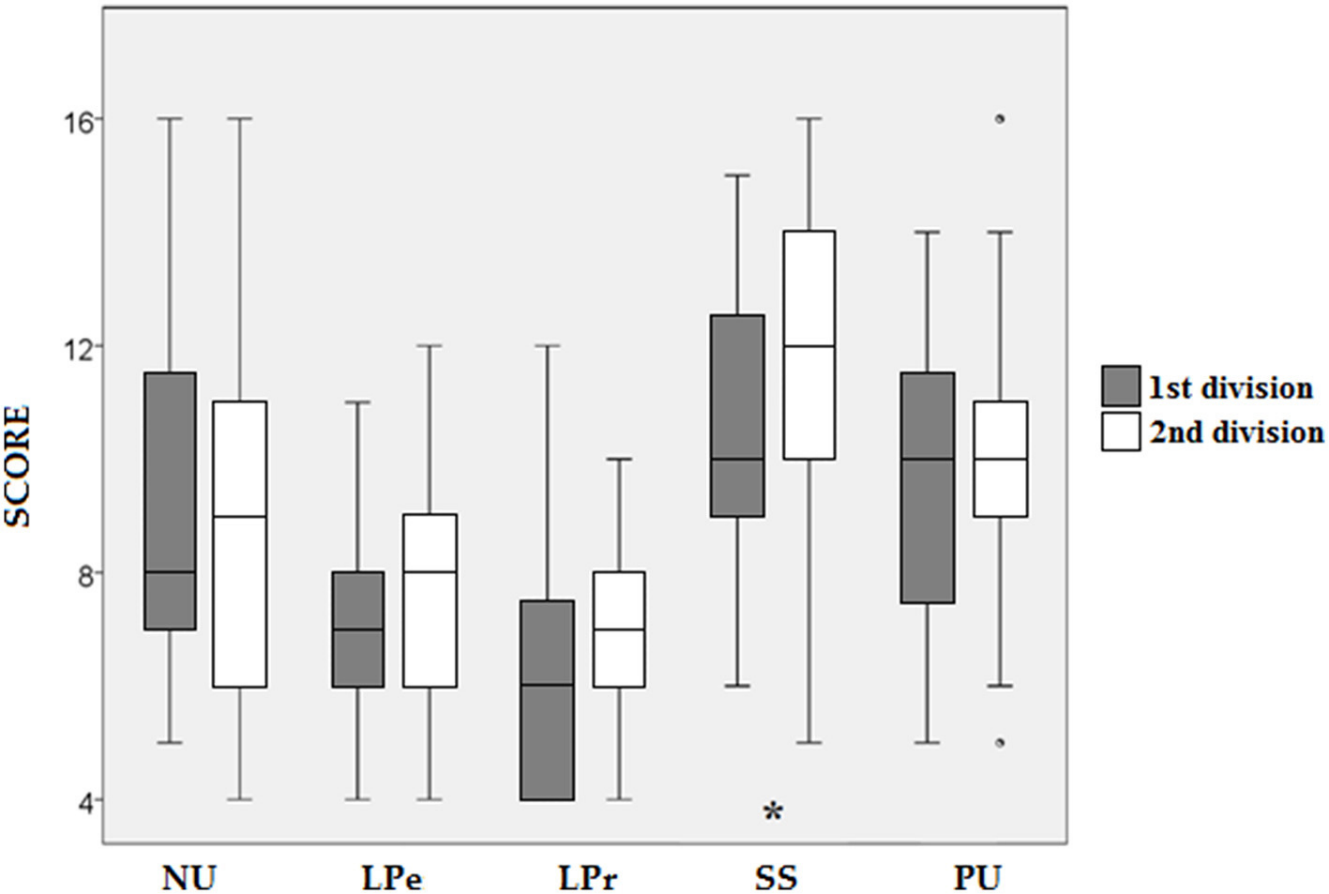
Comparison of the scores obtained in the dimensions of impulsivity according to the competition level. NU, Negative urgency; LPe, lack of perseverance; LPr, lack of premeditation; SS, sensation seeking; PU, positive urgency. *p < 0.05.

Table 5 shows the results of the Mann–Whitney test. Significant differences were found between genders in NU, with higher scores for women (*Z* = 2.480; *p* = 0.013; Figure 2) and small effect sizes (*r* = 0.283). Also, significant differences were found between competition levels in SS, showing second division players higher values (*Z* = 2.326; *p* = 0.02; Figure 3) and small effect size (*r* = 0.265). No other differences were found in neither impulsivity nor ER.

**Table 5:** Mann–Whitney test for the variables of the ERQ (reappraisal and suppression) and the UPPS-P (NU, LPr, LPe, SS, and PU), according to the gender and the competition level.

	**Sex**			**Competition level**		
	***Z***	***p***	***r***	***Z***	***p***	***r***
Reappraisal	1.203	0.229	0.137	0.027	0.979	0.003
Suppression	0.411	0.681	0.047	0.446	0.656	0.051
NU	2.480	0.013	0.283	0.306	0.760	0.035
LPr	0.742	0.458	0.085	0.873	0.383	0.099
LPe	1.781	0.075	0.203	1.155	0.248	0.132
SS	1.066	0.286	0.121	2.326	0.020	0.265
PU	0.376	0.707	0.043	0.384	0.701	0.044

## Discussion

The relationship between ER, impulsivity, and performance has been scarcely studied in a sports context. We hypothesized that these personality and emotional variables could be related to individual performance depending on the playing position, gender, and level of competition.

### Performance associations

Concerning performance, NU and SS correlated positively with the error ratio. Significant differences were found between playing positions in one dimension of impulsivity, namely NU, but not in the rest of the dimensions and ER. Likewise, female players presented higher NU than male players, and second division players showed higher SS than the top division players.

On correlating performance with the variables of ER and impulsivity, the error ratio was statistically associated with the NU and the SS. The results suggest that those players with a higher NU and SS made more mistakes when performing their serves and attacks. These findings follow the same tendency as those obtained by Castillo-Rodríguez et al. (2018), who also found associations between impulsivity and performance. However, their performance indicators were scored goals and assist in futsal, which correlated positively with SS. Previously, Lage et al. (2011) had also found a relationship between impulsivity and performance in team sports (i.e., handball), using, however, a different assessment test (Patton et al., 1995). Their results suggest an association between non-planning impulsivity (decision-making errors) and technical faults, between attentional impulsivity (omission errors) and fouls, and between motor impulsivity (commission errors) and rebounds with defense ball possession (negatively).

### Differences between playing positions

Concerning ER, elite volleyball players do not show differences according to their playing positions, which means that players tend to use both ER strategies, reappraisal, and suppression, independently of their playing position. A possible explanation is that, as happens with university athletes (Uphill et al., 2012), they might be using both reappraisal and suppression altogether to manage their emotions. Nevertheless, when studying a professional team-sport sample (hockey, basketball, soccer, and handball), Granado et al. (2014) discovered a prevalence of reappraisal over suppression when facing adverse match status. These results could lead to assuming that in elite volleyball players, these differences could exist as well, but our data did not suggest such differences. A possible explanation could be that Granado et al. (2014) associated the ER strategies with the type of emotion experienced in unfavorable situations, which our study did not associate.

There are differences between playing positions regarding impulsivity. To the best of our knowledge, no previous research has been performed on volleyball athletes regarding impulsivity. We consider this factor important because volleyball is an optimal sport for the study of impulsivity (González-Hernández et al., 2019). However, other sports have dealt with this matter. As in this study, previous study by Castillo-Rodríguez et al. (2018) found differences between playing positions in 111 elite futsal players. Offensive players presented higher general impulsivity scores than defensive players. A significant difference was found between goalkeepers and universals in SS. In this study, wing spikers presented higher NU than setters and middle blockers and liberos higher than middle blockers. The players with the highest NU were liberos and wing spikers, whereas setters and middle blockers presented the lowest values in NU. This might have to do with the expected result of the actions they perform; liberos and wing spikers are responsible for reception and attack, being both of their actions expected to be successful. Therefore, an error is quite evident. Setters and middle blockers are responsible for setting and blocking, respectively. In advanced levels, a setting mistake is perceived more in the consequent unsuccessful attack than in the set itself, whereas it is assumed that the block action is disadvantageous concerning the attack (Lima et al., 2019), and a few block points per match is considered a good performance (Millán-Sánchez et al., 2015).

### Differences between gender and competition level

Concerning gender, according to the results, there are no differences in any of the independent variables except for one dimension of impulsivity in Spanish volleyball players; female players presented significantly more NU than male players, with a small effect size (Cohen, 1988). Similar research has been previously conducted on sports samples showing results contrary to ours. For instance, a study with a sample of 62 Slovenian Olympic athletes (Kotnik et al., 2012) and another study with 34 elite junior judo athletes (Ferreira et al., 2017) presented no differences in impulsivity concerning gender in any dimension. It must be taken into account that judo is an individual sport in contrast with volleyball, whereas the study with Olympic athletes does not state what disciplines they perform (i.e., individual or team sports). Other than NU, neither dimension of the impulsivity showed statistical differences nor did the ER (reappraisal and suppression) variables.

Furthermore, these judo athletes presented a mean of 18.5 (males) and 18.9 (females) years old, compared to our 24.4 mean year-old sample. Age plays an essential role in impulsivity, as it decreases between adolescence and adulthood (Steinberg et al., 2008), and it might be affecting these different results. More recently, a study performed on a non-athlete sample showed higher impulsivity for men or women depending on the dimension (Wypych et al., 2018); men presented higher LPe and SS, whereas women had higher NU. This result in non-athlete participants is in line with ours on the volleyball sample. The NU refers to acting impulsively under negative emotions. Applied to volleyball, the NU might appear in the players after a succession of individual mistakes or when facing a negative match status. The results show that elite female volleyball players tend to react more impulsively than men in adverse conditions, contrary to what a biological explanation might reflect (Cross et al., 2011). This is an interesting result that must direct volleyball coaches in three directions. In the first place, they should identify the critical moments to use the appropriate game-stopping tools (i.e., time-outs and substitutions). Second, they should try to decrease their players' emotional pressure by downplaying the importance of the game situation and transmitting trust in their abilities. As a final resource, coaches ought to reorient the players' attention focus away from the emotional state and toward the game plan by making them recall some of its aspects to participate in the attention focus reorientation actively. Given these findings, this kind of intervention has a higher relevance in female players than in male volleyball players, for whom it could also be necessary, although they seem to cope with adverse emotional circumstances better than female players.

Moreover, the study compared volleyball players from two near competitive levels. Significant differences were found in one dimension of impulsivity, namely SS, with second division players presenting higher values than first division players. This is an interesting finding, despite the effect size being small (*r* = 0.265) (Cohen, 1988). No other variables presented differences. A systematic review comparing SS between athletes from different disciplines concluded that SS should not be used to explain the differences between elite and sub-elite groups (Gomà i Freixanet et al., 2012). Other studies comparing impulsivity between proficient and beginner athletes (not from the same discipline) found no differences (Thomson and Carlson, 2014). Given these results, the differences between competition levels were not expected. To the best of our knowledge, no research has dealt with comparing two competitive levels as close to each other as the top two national divisions of a single discipline. This fact might explain the differences found in this research.

Furthermore, second division players presented a slightly younger mean age than first division players, although the age difference does not influence the result (Steinberg et al., 2008). According to the findings presented, it would appear that players of a professional higher category might be less likely to take offensive risks. Previous research on volleyball match analysis has shown the influence of error on the result. The total number of errors increases in lost sets compared to won sets (Melendez-Nieves et al., 2020), and higher error ratios represent worse final rankings throughout the season (Drikos et al., 2009). Therefore, first division players might avoid unnecessary in-game mistakes that would be costlier than at the lower competitive level. Generally, SS refers to searching for new experiences; in a sports context, it represents a trend toward generating offensive advantages rather than keeping defensive stability (Gréhaigne et al., 2011; Castillo-Rodríguez et al., 2018). According to this, volleyball coaches should adjust the game model construction based on the offense or defense depending on the competition level (i.e., in second division teams, it is more recommendable to start the tactic system from the offensive structure). On an individual approach, it is suggested that volleyball players take the UPPS-P in order for their coaches to be aware of their results and be able to individualize the training process and favor those aspects for which there is a higher predisposition.

In summary, the results indicate that gender and competition level are important factors when examining emotion regulation strategies and impulsivity among volleyball players. Male players use reappraisal more frequently than female players, while female players use suppression more frequently. Similarly, players from the first division tend to rely more on suppression. In contrast, players from the second division tend to use reappraisal more frequently. These findings are consistent with previous research demonstrating gender and competition-related differences in emotion regulation and impulsivity. Specifically, studies by Gross and John (2003) and Cross et al. (2011) have found that male players are more likely to use cognitive reappraisal and score higher on measures of impulsivity than female players, respectively. Our study adds to the literature by highlighting the importance of considering these factors when designing interventions to improve emotional regulation and reduce impulsivity in athletes.

## Conclusion

The volleyball players from the top two Spanish divisions differ in NU according to playing position and gender. Wing spikers present higher NU than setters and middle blockers, whereas liberos also show higher NU than middle blockers. Female players display higher NU than their male counterparts. In addition, second division players present a higher SS than the players in the top division. Finally, higher values in NU and SS represent higher error ratios overall.

These results according to playing position, gender, and competition level show that players differ in emotional aspects, such as some dimensions of impulsivity, that might influence how they approach their game. Practitioners might use the available tools (i.e., questionnaires) to know their players' emotional traits and design training processes accordingly.

## Practical applications

Identifying the factors that affect athletes' performance is essential for developing effective interventions. In the case of volleyball, training in technical and physical skills is crucial for optimal player performance. However, it has also been demonstrated that players' ability to regulate their emotions and manage impulsivity is critical for success on the court. Therefore, coaches and therapists could work with volleyball players to develop effective emotion regulation and impulsivity reduction strategies. For example, visualization techniques could help players manage their anxiety before an important match. A mindfulness training program could also enhance players' ability to stay present-focused and reduce impulsivity in high-pressure situations. These interventions can improve players' performance and emotional wellbeing, which can positively impact the team as a whole.

## Limitations and future research

This study's sample is composed of professional and highly specialized players, with everything that means concerning the possibility to be accessed and studied. An increase in the sample size might cause a decrease in the value of the result, in terms of specificity and competitiveness (Lago Peñas et al., 2020), and frequently, studies similar to ours also had a small sample (Ferreira et al., 2017; Castillo-Rodríguez et al., 2018; Montuori et al., 2019). The authors acknowledge that the sample size represents a formal limitation, even more, when it has been necessary to divide it to perform the analyses according to the playing position. As a consequence, the results must be interpreted with caution, awaiting future studies with bigger sample sizes to check whether these trends consolidate. Further research should carry out similar analyses in younger players to compare results with those of an elite sample. Finally, the specialization of the volleyball players makes the comparison based on their game actions somewhat complicated; some of them do not perform certain actions by regulation, whereas others are not responsible for other actions because of their playing position. Therefore, future studies ought to consider deeper performance indicators that contemplate a way to compare players regardless of these factors.

## Data availability statement

The raw data supporting the conclusions of this article will be made available by the authors, without undue reservation.

## Ethics statement

The studies involving human participants were reviewed and approved by Comité de Ética en Investigación de la Universidad de Granada. The patients/participants provided their written informed consent to participate in this study.

## Author contributions

AM-S, DC, RV, and AU contributed to the conception and design of the study. RV provided expert background. AM-S collected the data. IM and AM-S performed the data analysis. AM-S and AU wrote the first draft of the manuscript. All authors revised and edited the different versions of the manuscript and approved the submitted version.
